# Effectiveness of Local Glucocorticoid Infiltration Versus Traditional Gauze Bandaging for the Treatment of Onychocryptosis: A Randomized Controlled Trial

**DOI:** 10.3390/healthcare12212139

**Published:** 2024-10-27

**Authors:** María Teresa García-Martínez, Alfonso Martínez-Nova, Angélica María Fernández-Gómez, José-María Blasco, Francisco Vilchez-Márquez, Carmen García-Gomariz

**Affiliations:** 1 Department of Nursing, University of Valencia, Menéndez y Pelayo Av. 19, 46010 Valencia, Spain; maria.t.garcia-martinez@uv.es (M.T.G.-M.); carmen.garcia-gomariz@uv.es (C.G.-G.); 2 Department of Nursing, University Center of Plasencia, University of Extremadura, 10600 Cáceres, Spain; afernandaxj@alumnos.unex.es; 3 Department of Physiotherapy, University of Valencia, 46010 Valencia, Spain; jose.maria.blasco@uv.es; 4 Group of Physiotherapy in the Ageing Process: Social and Health Care Strategies, Department of Physiotherapy, Universitat de València, Calle Gascó Oliag 5, 46010 Valencia, Spain; 5 Hospital Virgen de las Nieves, 18014 Granada, Spain; fvilchezm@hotmail.com; 6 Group of Research Advances in Ankle and Foot, Department of Nursing, University of Valencia, Menéndez y Pelayo Av. 19, 46010 Valencia, Spain

**Keywords:** onychocrytopsis, conservative treatment, health care, local infiltration, glucocorticoid infiltration, gauze bandaging

## Abstract

Background/objectives: Local intralesional corticosteroid injections into the periungual fold are used to treat patients with onychocryptosis, but their short- or medium-term effects are unknown. The goal was to compare the efficacy of this treatment in stages IIa, IIb, and III of the condition with a common conservative method such as gauze bandaging. Methods: A two-arm randomized trial with 40 patients with stage IIa, IIb, and III onychocryptosis equally assigned into two treatment groups—control (spiculotomy and application of gauze bandaging) and experimental group (spiculotomy and infiltration of corticosteroid)—was performed. Anthropometric data, initial clinical status, pain, and inflammatory measures were collected before and at least one month after the intervention. Results: Pain reduction was higher in the experimental group (5.5 vs. 4.8 points) but with no significant differences (*p* = 0.306).Corticosteroids significantly reduced inflammation over gauze bandaging (1.9 vs. 1) with significant differences between them (*p* = 0.029). Conclusions: Corticosteroid infiltration was more effective than gauze bandaging application in reducing inflammation in patients with onychocryptosis, with similar effects on pain. These findings support the clinical importance of corticosteroid treatment for this condition, although a single infiltration may not be sufficient to prevent relapses.

## 1. Introduction

Onychocryptosis is an alteration of the nail apparatus in which the nail plate injures the lateral fold due to the continuous trauma that it causes during its growth, with symptoms such as pain, inflammation, functional limitation, and sometimes a pyogenic infectious condition [1]. It is mainly located in the first toe, although it can be found in the minor toes in isolation or even coexisting in several toes at the same time [1,2]. Approximately 20% of the population suffers from this pathology, being more frequent in adolescents and young adults [3]. Some authors claim that it occurs with a greater prevalence in men than in women, while most authors agree that this pathology is more common in men with a 3:1 predominance compared to women, especially in the age period between 8 and 40 years old. It has been observed that from the third decade of age onwards, the frequency of this pathology does not discriminate between sexes and has a higher percentage of involvement in the first toe [4].

The severity of the injury and possible treatment are usually determined by the size and hypertrophy of the lateral folds [2,3]. In agreement with Mozena and Martinez Nova [1], conservative treatments are effective in reducing painful processes in the early stages of the pathology. In case of recurrence, nail growth is re-educated through spiculotomy (cutting the ingrown portion of the nail), gauze bandaging, nail re-conduction with acrylics, plastic or metal orthonyxia, or a combination of treatments [5,6]. However, these techniques have relatively high recurrence rates, from 80% for simple spiculotomy to 40% for gauze bandaging [4]. Other acrylic nail reshaping techniques have a relatively low recurrence rate of 10%, although it does not appear to be useful in patients with nail fold inflammation or granuloma [7]. 

Intralesional corticosteroid infiltrations have been used in dermatology for different nail disorders, such as retronychia [8,9,10], nail psoriasis [11,12], lichen planus [13], or even onychocryptosis [14,15]. The main anti-inflammatory effect is achieved right where the nail causes the most inflammation [16,17]. We have found few studies analyzing perilesional triamcinolone as a treatment for foot onychocryptosis. The first addressed five cases, with 100% success and no lesion recurrence between 6 months and 3 years and a single dose [14]. In addition, Wang et al. [15] reported success rates of 87% in stages I and IIa and 66% for stages IIb and III; however after 6 months the patients exhibited reduced inflammation but still presented symptoms of onychocryptosis. Moreover, patients experienced symptomatic and temporal improvement. The recurrence was present because there were external factors to correct such as biomechanics or footwear. However, they did have a lower stage of grade 1, and none of them were grade 2 or grade 3 again [15].

These investigations suggest that intralesional infiltration of corticosteroids along the entire affected nail fold may be effective for stage I and IIa onychocryptosis, being a possible treatment for symptomatic relief in stages IIb and III for the comprehensive health care of patients or in those who cannot undergo surgery [15]. However, the efficacy of corticosteroid injection compared to other conservative methods has not yet been studied. Thus, this study aimed to compare the reduction in pain and inflammation of the nail fold in two groups (corticosteroid vs. gauze bandaging) after one month of treatment.

## 2. Materials and Methods

### 2.1. Procedures and Participants

This is a two-arm randomized trial in which participants were randomly assigned into one of two groups: the control group, which received gauze bandaging and spiculotomy; or the corticosteroid group, which received corticosteroid infiltration and spiculotomy. The study was conducted at the Aquilesia Podiatric Center in Vila-Real (Spain). 

Overall, 40 patients with onychocryptosis, aged 31.5 (SD 17) years, IMC 23.8 (SD 4.3 kg/m^2^) (see Table 1), took part. (1) Patients who voluntarily requested treatment for their pathology in the participating centers were included when (2) diagnosed with onychocryptosis (3) and presented with inflammation in stages IIa, IIb, and III. Patients were excluded if (1) they were under pharmacological treatment that interacted with anesthesia or corticosteroids, (2) had trypanophobia, (3) were allergic to anesthesia or corticosteroids, (4) had paronychia, or (5) had onychocryptosis caused by increased curvature of the nail plate and without inflammation in the lateral fold.

The study complied with the requirements of the Declaration of Helsinki, being approved by the Bioethics and Biosafety Committee of the University of Extremadura (ID: 198A//2020 and 184//2023) and registered in ClinicalTrials.gov (NCT05214586). Patients participated voluntarily in the study and signed a consent form.

### 2.2. Randomization and Masking

An assistant from the podiatric clinic referred the initial examinations to a member of the research team who made the diagnosis to screen the sample that met the inclusion criteria. A binomial random sequence was generated by the biometrician of this study by software (Matlab^®^ 9.13) in Origin. Participants were sequentially assigned by the principal investigator following the sequential output of such a sequence. The therapist in charge of interventions was informed about participant allocation by the principal investigator and then, the participants were scheduled to undergo the interventions. No information about group assignment was given to participants. Despite this procedure, blinding of participants could not be ensured, since they were originally informed about the possible interventions and somehow could be aware of the differences between both treatments. A podiatrist with more than 10 years of experience oversaw the interventions. All the interventions were delivered by such a member of the research team. A member of the team, blinded to the interventions, was in charge of extracting and processing the data, which were previously anonymized with numerical codes.

### 2.3. Interventions

All participants underwent spiculotomy. The affected area was disinfected with chlorhexidine, a powerful and fast skin disinfectant [18,19,20]. The spicule was removed by cutting the segment of the nail plate embedded in the periungual fold. The procedure was conducted with pliers, Adson forceps, and a scalpel with a number 15 blade. 

**Figure 1:**
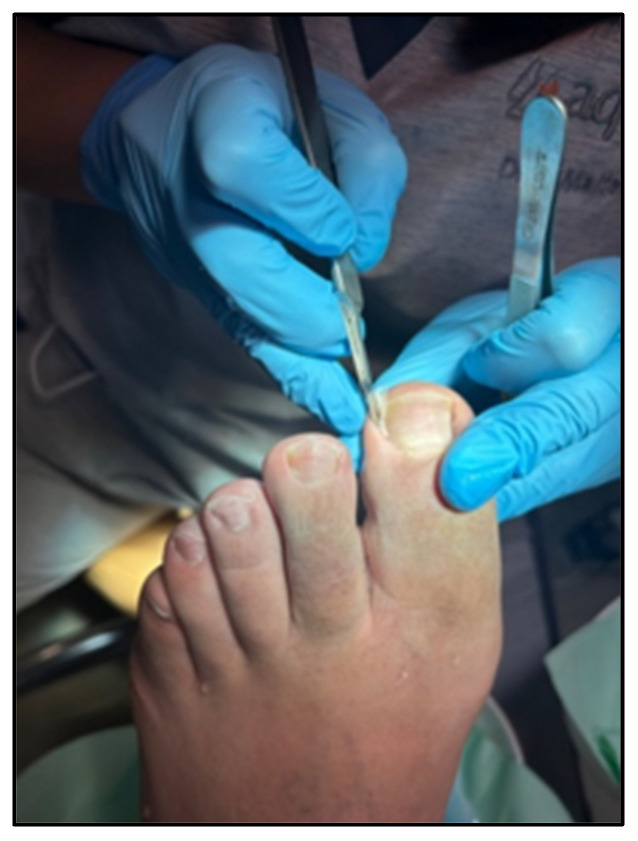
Mulched with gauze bandaging after spiculotomy.

**Figure 2:**
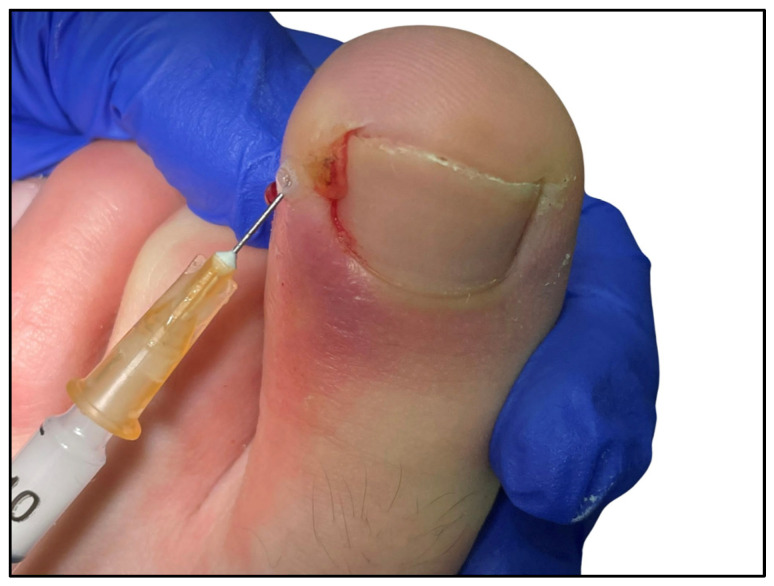
Infiltration of the anesthetic + corticosteroid solution, sweeping along the lateral fold.

Then, gauze bandaging was applied to participants in control group [7] by placing a small piece of gauze soaked in povidone iodine or chlorhexidine under the nail, using a gouge or scalpel and tweezers [5] (see Figure 1). However, the participants in the experimental group received a corticosteroid infiltration. Betamethasone was used for this treatment. Once the area was disinfected, the nail segment embedded in the lateral fold was removed. Then, the corticosteroid was injected intralesionally over the area of maximum inflammation with 0.5 cc of Betamethasone combined with 0.5 cc of 2% Mepivacaine. This process was carried out with an insulin needle, with a single puncture depositing the solution along the entire inflamed and hypertrophied lateral fold as the needle was withdrawn (Figure 2). It is recommended not to reach the hypodermis, which is where the fat susceptible to atrophy is located. Deposit the preparation in the epidermis and dermis. To prepare the injection, it is recommended to previously load the corticosteroid into the anesthesia so that the first drops in contact relieve the infiltration. Cold spray was also used when the needle was locked.

### 2.4. Measures and Timeline

Personal data, weight, height, body mass index, type of technique used (gauze bandaging or corticosteroid), gender, affected foot, and affected canal were collected. There were no baseline differences between weight, height, BMI, age, and initial pain and inflammation (*p* > 0.05, Table 2).

**Figure 3:**
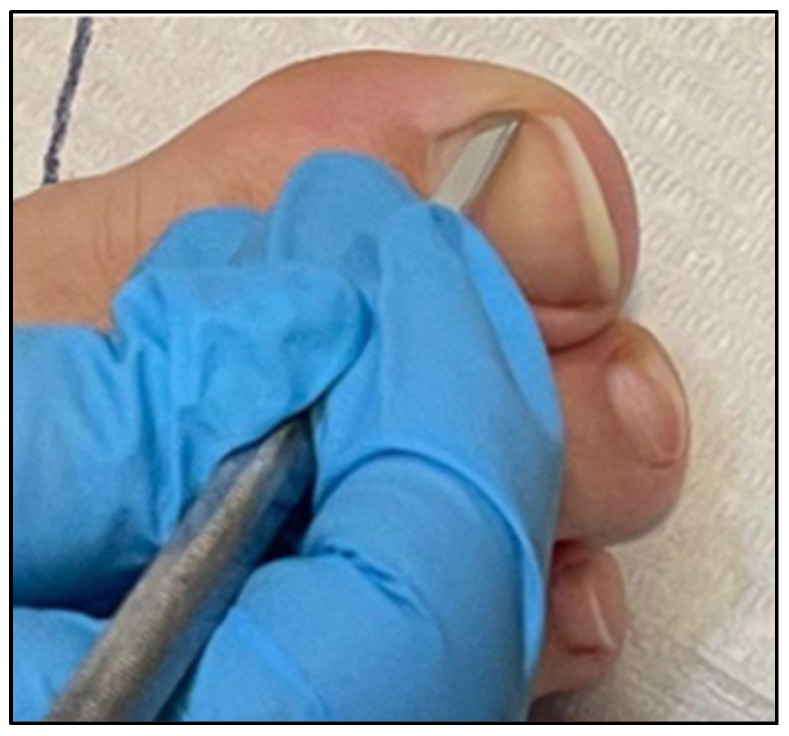
Introduction of a measuring instrument to assess the amount of impeller affecting the nail plate.

**Figure 4:**
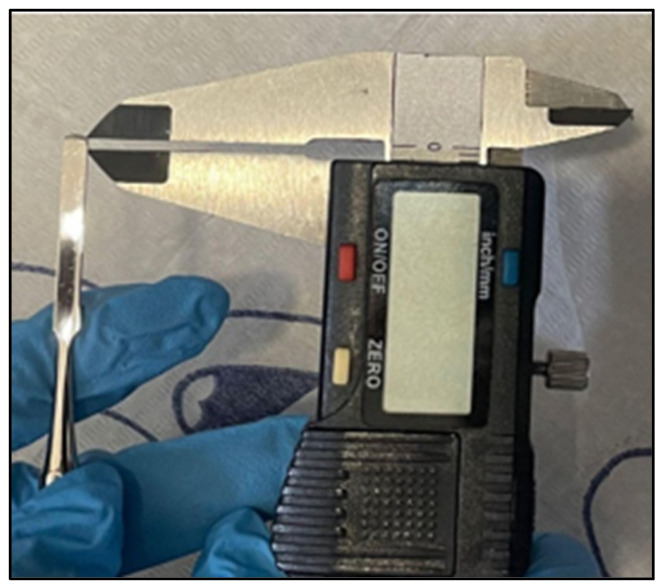
Precision caliper impeller depth measurement.

Regarding clinical measures, pain and inflammation were assessed. Pain was assessed with a visual analogue scale (VAS) in a smartphone app [21] where the patient was shown the VAS on a smartphone and with a pointer had to indicate his/her level of pain, where the labels of the end points on the left and right sides of the horizontal line indicated “no pain” and “worst possible pain”, respectively. The patient marked the level of intensity of the pain caused by his/her pathology on the screen of an electronic device, taking the average as a reference value. The inflammation of the lateral fold was assessed with a digital caliper and a straight metal spatula. The spatula was carefully introduced between the lateral fold and the nail plate (Figure 3). Then, the distance from the edge of the spatula to the lateral fold was accurately measured (Figure 4).

A baseline assessment of pain and inflammation was performed before the treatments. One week after the treatment, a first check-up assessed possible complications. One month after the intervention, the baseline measurements were repeated. Finally, the level of satisfaction with the procedure and possible recurrences were collected.

### 2.5. Data Analysis

A descriptive analysis of the sample characteristics and inferential analyses were performed to assess differences between groups and to test the hypothesis. Distribution tests were performed independently in each group to determine whether the parameters evaluated followed a normal distribution using Kolmogorov–Smirnov and Shapiro–Wilk tests. Possible baseline differences were checked with Student’s *t*-tests and their corresponding non-parametric or Chi-square tests for continuous or qualitative variables, respectively.

To test the hypothesis and see if there were differences between groups in the effects on the reduction of pain and inflammation, the Student’s *t*-test or the corresponding non-parametric test was performed. Secondary analyses were performed to assess the within-group and between-group time effect with *t*-tests, and to assess differences between groups in level of satisfaction and recurrence, the Chi-square and *t*-tests were conducted. The SPSS version 23.0 program licensed by the University of Valencia was used. Confidence intervals were set at 95%.

## 3. Results

All participants (*n* = 40) had a pain reduction of 5.2 (SD 1.9) points. Although participants reduced their pain significantly, no treatment produced greater benefits (see Table 2) because there were no differences between using one or the other treatment in reducing pain. Similarly, all participants reduced the level of inflammation by 1.5 (SD 1.2) points, with significant differences after the treatments. However, in this case, participants in the experimental group did experience greater improvements, with statistically significant differences (*p* = 0.029) (see Table 2). Overall, the level of satisfaction was 7.05 (SD 2.2) after treatment, so all participants had a similar level of satisfaction, regardless of the intervention group. A total of 42.1% of participants in the control group suffered relapse compared to 38.1% of the experimental group, but without statistical insignificance (*p* = 0.796).

## 4. Discussion

This trial found that patients with onychocryptosis treated with a spiculotomy, plus either a gauze bandaging or a corticosteroid injection, improved their clinical status regarding pain and inflammation. However, corticosteroid infiltration reduced inflammation to a greater extent. The reduction in pain in the control group may be because spiculotomy and gauze bandaging help to separate the nail from the periungual tissue, which may provide some mechanical relief; however, this treatment does not have the active pharmacological properties to reduce inflammation and pain that the corticosteroid did. 

In their study, Wang et al., were presented with an unwanted side effect, the atrophy of fat in the infiltration area, which disappeared after months. In our study, some patients reported greater sensitivity in the affected area, but we cannot say if it was the injection or the persistent inflammation of the pathology [15].

Intralesional infiltration of the corticosteroid directly injected into the injured area of the lateral fold may explain the greater reduction in inflammation since the concentration of the medication in the damaged area had a more intense and rapid effect [10]. However, the benefits of the corticosteroid are focused solely on the effects on inflammation. All subjects had a high level of satisfaction with the procedure performed, although there was a 42.1% recurrence in the gauze bandaging group and 38.1% in the corticosteroid group. These results show us that neither of these two treatments is definitive for the solution of onychocryptosis, although the corticosteroid seems to present an advantage, reducing the inflammation of the affected nail fold and exhibiting a great reduction in the granuloma, which could lead to avoiding the nail becoming ingrown in the future by reducing the fibrosis of the lateral fold [15]. 

The results suggested that a single injection does not seem to be useful in preventing recurrent onychocryptosis. The results of our study did not reach the same level of success as those of the previously published study with five cases [14], where the recurrence rate was zero. It is important to note that, unlike our approach, Vilchez et al. [14] used multiple punctures and prolonged the follow-up period more than in this study. Another study with a larger sample size [15] reported better results in patients with lower stages (stage I, IIa, IIb, and III were 88.89%, 86.36%, 66.67%, and 66.67%, respectively), but after 6 months the patients had relapses, with less inflammation but with the initial pathology despite adding gentamicin, a powerful antibiotic, to the injection and prescribing infiltrations every week if the pathology had not remitted. In our study, it is observed that by performing a single infiltration there is a reduction in inflammation, reduction in the granuloma, and reduction in pain in both treatments, so they would be equally valid, and there were fewer recurrences in patients with the experimental treatment (38.1%) compared to the control group (42.1%). In future studies, we should also differentiate between hypertrophy of the lateral fold and granuloma. In our study, we considered it as a common sign of onychocryptosis and therefore as a single item, but in our experience, the granuloma disappeared and the lateral fold was reduced. 

Furthermore, in our study, there were no unwanted side effects, as reported by Wang et al. [15], who described three patients with skin atrophy at the injection site that disappeared over time.

The study shows promising future applicability, as corticosteroid treatment was more effective in reducing inflammation, potentially improving podiatry protocols. The findings open new opportunities for combining conservative treatments, offering potential to reduce recurrence rates, and it could become a tool for the podiatrist in those patients who cannot access definitive treatment at that time for sports, work, or economic reasons. We believe that together with the biomechanical control of the toe and the increase in injections, we can correct the deformity in certain onychocryptoses, as long as footwear or bad cut is not the origin.

Still, some limitations in this study must be acknowledged. The results should be treated cautiously; for instance, the recurrences were estimated considering any patient who presented minimal symptoms, even if their clinical condition was better than at the beginning of the treatment. Only a single injection was performed; more injections—as the other studies performed—could have improved the recurrence rates and pain to a greater extent; however, this must be studied exhaustively and is only speculation. For future research, it would be beneficial to carry out a long-term follow-up to evaluate the durability of the results. Also, it would be beneficial to consider other methodologies such as combining corticosteroid and gauze bandaging, or oral or topical antibiotic therapy. In addition, it would be beneficial to explore other aspects, such as footwear, activities or work performed, and knowledge of basic care such as nail cutting.

Injections with corticosteroid present an economic increase compared to a conventional conservative treatment such as spiculotomy or gauze bandaging. However, it is cheaper than surgery. It offers patients with surgical indications to reduce inflammation by reducing the surgical procedure or offering more time if it cannot be treated invasively at that moment.

Furthermore, studying and exploring the effects of repeated corticosteroid injections, as well as comparing the results between different corticosteroids or formulating the injection with an antibiotic like Wang X et al., or combining corticosteroid therapy with other conservative measures such as orthonyxia or nails, could pave the way for future lines of research and include these options in future studies, providing a more complete treatment strategy. 

## 5. Conclusions

This study supports that intralesional infiltration with a corticosteroid is more effective in reducing the inflammation associated with onychocryptosis than gauze bandaging. However, it does not produce greater benefits in reducing pain, or in other aspects such as patient satisfaction or improving the recurrence rate. However, it could become a valid therapeutic option in a podiatrist’s treatment plan, especially if the frequency of infiltrations is increased or combined with other therapies.

## Figures and Tables

**Table 1:** Baseline sample characteristics.

	Experimental Group (*n* = 21)	Control Group (*n* = 19)	*p*-Value
**Weight (kg)**	66.04 (17.52)	63.05 (12.17)	0.538
**Height (m)**	1.65 (0.09)	1.63 (0.10)	0.556
**BMI (kg/m^2^)**	23.96 (4.67)	23.62 (4.00)	0.812
**Age (yrs.)**	28.67 (15.79)	34.63 (18.54)	0.283
**Pain (0–10)**	7.15 (0.94)	6.75 (1.58)	0.339
**Inflammation (0–10)**	3.25 (1.26)	2.61 (1.12)	0.101

Data are given as means (SD).

**Table 2:** Results of intervention.

	Experimental Group (*n* = 21)		Control Group (*n* = 19)		Intra-Group (*n* = 40)	*p*-Value ^+^		*p*-Value * (Time)
	AI	BC	AI	BC	BC	AI	BC	0.0000
**Pain (0–10)**	1.55 (1.9)	5.49 (1.81)	1.86 (1.6)	4.84 (2.16)	5.18 (1.9)	0.597	0.306	
**Inflammation (0–10)**	1.67 (1.6)	1.95 (1.01)	1.52 (1.0)	1.08 (1.31)	1.54 (1.23)	0.537	0.029	

Notes: AI = after intervention; BC = baseline change (reduction); (^+^) inter-group; (*) intra-group time.

## Data Availability

The original contributions presented in the study are included in the article, further inquiries can be directed to the corresponding author.
